# Pancreaticoduodenectomy for biliary tract carcinoma with situs inversus totalis: difficulties and technical notes based on two cases

**DOI:** 10.1186/1477-7819-11-312

**Published:** 2013-12-17

**Authors:** Daisuke Kyuno, Yasutoshi Kimura, Masafumi Imamura, Motonobu Uchiyama, Masayuki Ishii, Makoto Meguro, Masaki Kawamoto, Toru Mizuguchi, Koichi Hirata

**Affiliations:** 1 Departments of Surgery, Surgical Oncology and Science, Sapporo Medical University School of Medicine, 1-South, 16-West, Chuo-ku, Sapporo 060-8543, Japan

**Keywords:** Biliary tract carcinoma, Cardiovascular malformation, Hepatobiliary malformation, Pancreaticoduodenectomy, Situs inversus totalis

## Abstract

Situs inversus totalis (SIT) denotes complete right-left inversion of the thoracic and abdominal viscera. Diagnosis and surgical procedures for abdominal pathology in patients with SIT are technically more complicated because of mirror-image transposition of the visceral organs. Moreover, SIT is commonly associated with cardiovascular and hepatobiliary malformations, which make hepatobiliary-pancreatic surgery difficult. Two cases of pancreaticoduodenectomy for biliary tract carcinoma in patients with SIT are presented. Both patients had an anomaly of the hepatic artery. Advanced diagnostic imaging techniques were very important for careful preoperative planning and to prevent misunderstanding of the arrangement of the abdominal viscera. This facilitated the surgical team’s adaptation to the mirror image of the standard procedure and helped avoid intraoperative complications due to cardiovascular and hepatobiliary malformations associated with SIT. Pancreaticoduodenectomy in patients with SIT can be performed successfully with detailed preoperative assessment, use of effective techniques by the surgeon, and appropriate support by assistants.

## Background

Situs inversus totalis (SIT) is characterized by a left-to-right reversal of the abdominal viscera with dextrocardia. This is in contrast with situs inversus viscerum (also termed situs inversus, SI), which means a complete mirror-image transposition of the abdominal visceral organs with normal orientation of the thoracic organs. Situs inversus totalis is found in 1/8000 to 1/25,000 of the normal population [1]. Generally, this rare anomaly is discovered or diagnosed incidentally during thoracic and abdominal imaging. Although the exact etiology of SIT remains unknown, SIT is thought to result from chromosomal abnormalities that lead to a reversal of right-left polarity [2-4].

Diseases associated with SIT include bronchiectasis and chronic sinusitis with ciliary immotility (Kartagener syndrome) [5,6]. Patients with SIT have an increased risk of cardiac, splenic, and hepatobiliary malformations [7]. These abnormalities are not considered to be premalignant, and SIT in itself does not seem to affect health or life expectancy.

Diagnosis and surgical procedures for abdominal pathology in patients with SIT are technically more difficult because the anatomical positioning of the visceral organs is arranged differently [8,9]. Previous authors have reported performing pancreaticoduodenectomy in patients with SI or SIT [10-15]. They reported that the surgical procedures were difficult because of the anatomical abnormalities and that special attention should be paid to diagnosis and preoperative staging. Therefore, on the basis of these reports, more detailed and advanced information is required for surgeons. Taking into account experience with past cases of successful pancreaticoduodenectomy for biliary tract carcinoma with SIT, this report focuses on the difficulties and technical notes of pancreaticoduodenectomy in patients with SIT.

### Surgical procedure and techniques in patients with SIT

Pylorus-preserving pancreaticoduodenectomy with modified Child’s reconstruction is our standard procedure. The key parts of the common surgical procedures in patients with SIT in these two cases were as follows:

1. Detailed preoperative assessment with advanced imaging studies was helpful to detect anatomical abnormalities associated with SIT and prepare the plan for the procedure.

2. The right-handed surgeon stood on the right side of the patient, as usual.

3. To smoothly perform the Kocher maneuver from left to right, the surgeon provided countertraction, and the first assistant mobilized the duodenum and the head of the pancreas.

4. Each surgical procedure was performed while encircling the major vessels (the superior mesenteric vein (SMV), portal vein (PV), common hepatic artery (CHA), gastroduodenal artery (GDA), proper hepatic artery (PHA), and the right and left hepatic arteries) with tapes. The major vessels were identified as landmarks to avoid any errors, and the operation was performed in the same order as the standard procedure.

5. The procedures around the superior mesenteric artery (SMA) plexus and the inferior pancreaticoduodenal artery (IPDA) were difficult. The surgeon changed his position to achieve adequate countertraction between the uncinate process of the pancreas and the SMA-plexus.

6. To adequately suture the pancreatic duct to the jejunal mucosa for the pancreaticojejunostomy, the surgeon changed his position or used double-armed sutures. Hepaticojejunostomy (end-to-side) and duodenojejunostomy with Braun’s anastomosis were performed as usual.

7. Two closed-system drainages were placed in the foramen of Winslow and around the pancreaticojejunal anastomosis, as in the standard procedure.

## Case presentations

### Case 1

**Figure 1:**
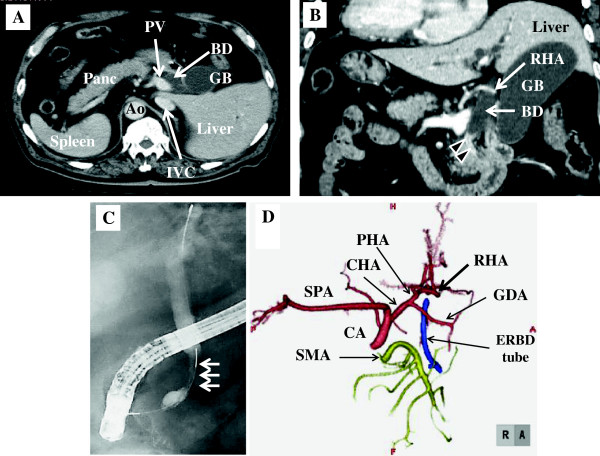
**Preoperative imaging of patient 1. (A)** Contrast-enhanced CT scan and **(B)** coronal image of multidetector-row CT reveal a dilated bile duct. **(C)** Endoscopic retrograde cholangiography shows distal bile duct stenosis (arrows) and upstream dilation of the bile duct. **(D)** Three-dimensional angiography with multidetector-row CT shows a left-to-right reversal of the vascular arrangement, but does not show vascular malformation. Ao, aorta; BD, bile duct; CA, celiac artery; CHA, common hepatic artery; ERBD tube, endoscopic retrograde bile duct drainage tube; GB, gallbladder; GDA, gastroduodenal artery; IVC, inferior vena cava; Panc, pancreas; PHA, proper hepatic artery; PV, portal vein; SMA, superior mesenteric artery; SPA, splenic artery.

A 74-year-old man with SIT was referred to our hospital for investigation and treatment of distal bile duct carcinoma. His past medical history included hypertension and cerebral hemorrhage. A chest X-ray showed dextrocardia. Enhanced computed tomography (CT) revealed a right-sided spleen and that the inferior vena cava (IVC) lay to the left of the aorta (Figure 1A). The head of the pancreas was to the left of the midline. Distal bile duct stenosis and upstream dilation of the bile duct were shown on coronal images of multidetector-row CT (MD-CT) and endoscopic retrograde cholangiography (Figure 1B,C). Biopsies of the distal bile duct identified an adenocarcinoma. Intraductal ultrasonography of the distal bile duct showed that the carcinoma had invaded the head of the pancreas. Enhanced CT did not show any distant metastases. Three-dimensional angiography with MD-CT showed that the right hepatic artery, which was the left-sided hepatic artery in this case, ran along the ventral side of the bile duct (Figure 1B,D).

**Figure 2:**
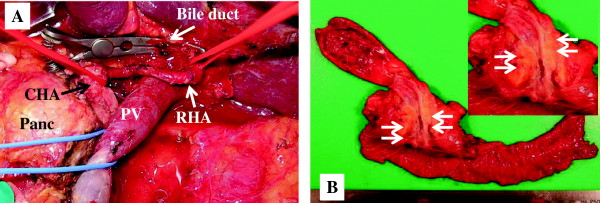
**Photographs of patient 1. (A)** Intraoperative photograph prior to reconstruction. **(B)** Macroscopic view of the resected specimen shows adenocarcinoma of the common bile duct (arrows) infiltrating the head of the pancreas. CHA, common hepatic artery; Panc, pancreas; PV, portal vein; RHA, right hepatic artery.

The patient underwent pancreaticoduodenectomy (Figure 2A). After dividing the neck of the pancreas, the positions of the SMV and the PV were taken as major landmarks to perform the procedure. The anomaly of the right hepatic artery had no significant impact on the surgical procedure because the operative procedure was planned based on the preoperative imaging studies.

The surgeon stood on the left side of the patient during excision of the SMA-plexus and the duct-to-mucosal anastomosis of the anterior row in the pancreaticojejunostomy. The procedures around the SMA plexus and the IPDA were performed with the following three steps:

1. The surgeon retracted the SMV and the PV with the left hand, and resected the pancreatic nerve plexuses from the caudal side.

2. The surgeon changed his position and obtained countertraction between the uncinate process of the pancreas and the SMA plexus with the left hand. He resected the pancreatic nerve plexuses from the cranial side.

3. After the jejunum was transected and the jejunal mesentery near the ligament of Treitz was divided, the head of the pancreas was passed underneath the SMV over to the right side of the abdomen. The pancreatoduodenum was retracted by the left hand, and the neural plexus connecting around to the SMA and the IPDA were divided.

The operation time was 564 min, and the intraoperative blood loss was 600 ml. Histopathological findings of the resected specimen confirmed the diagnosis of a well-differentiated adenocarcinoma of the common bile duct infiltrating the head of the pancreas, with three metastatic lymph nodes (Figure 2B). The biliary, pancreatic, duodenal, and retroperitoneal margins were free of tumor. The tumor was stage IIB with T3N1M0 according to the American Joint Committee on Cancer/Union for International Cancer Control (AJCC/UICC) TNM staging system [16]. The patient’s postoperative course was complicated by a pancreatic fistula (grade B according to the International Study Group of Pancreatic Fistula (ISGPF) definition) without any serious symptoms of infection. The patient was discharged after the leakage was ameliorated with drainage.

### Case 2

**Figure 3:**
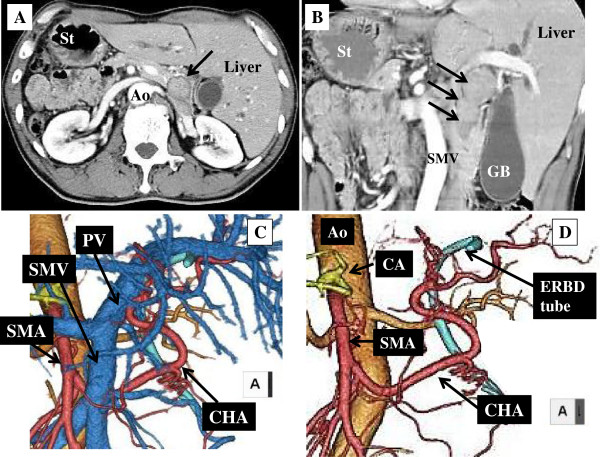
**Preoperative imaging study of patient 2. (A)** Contrast-enhanced computed tomography scan and **(B)** coronal image of multidetector-row CT show the tumor from the proximal to the distal portion of the bile duct (arrows). **(C,D)** Three-dimensional angiography with multidetector-row CT shows a left-to-right reversal of the vascular arrangement and that the common hepatic artery arises from the SMA and runs behind the SMV. Ao, aorta; CA, celiac artery; CHA, common hepatic artery; ERBD tube, endoscopic retrograde bile duct drainage tube; GB, gallbladder; PV, portal vein; SMA, superior mesenteric artery; SMV, superior mesenteric vein; St, stomach.

A 67-year-old man with no previous illnesses was referred to our hospital for investigation and treatment of bile duct carcinoma. Situs inversus totalis was first discovered at the referring hospital. Enhanced CT confirmed the transposition of viscera as follows: dextrocardia, right-sided stomach and spleen, left-sided liver, and IVC to the left of the aorta (Figure 3A). Computed tomography also revealed a dilation of the intrahepatic bile ducts, and a tumor from the proximal to the distal portion of the bile duct (Figure 3A and B). Distant metastases were not detected. Magnetic resonance cholangiopancreatography confirmed a tapered stricture of the bile duct with upstream dilation of the bile duct.

Three-dimensional angiography with MD-CT showed vascular anomalies. The entire hepatic artery did not arise from the celiac artery, but arose from the SMA. The hepatic artery ran behind the SMV. The artery ran along the ventral side of the pancreas and gave rise to the left and right hepatic arteries (Figure 3C,D). This type of arterial anomaly belongs to type VI of Adachi’s classification and type IX of Michels’ classification [17,18]. There was no evidence of any other anatomical anomalies.

**Figure 4:**
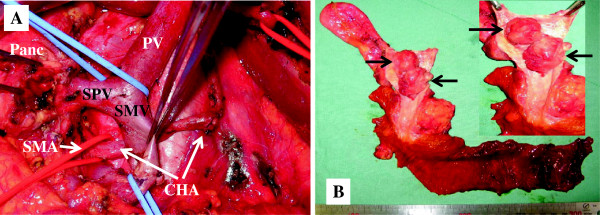
**Photographs of patient 2. (A)** Intraoperative photograph prior to reconstruction. **(B)** Macroscopic view of the resected specimen shows adenocarcinoma of the common bile duct (arrows). CHA, common hepatic artery; Panc, pancreas; PV, portal vein; SMA, superior mesenteric artery; SMV, superior mesenteric vein; SPV, splenic vein.

The patient underwent pancreaticoduodenectomy. The procedure was completed using the schematic diagram reconstructed from the preoperative three-dimensional CT imaging (Figure 3C,D). Before dividing the neck of the pancreas, the surgeon identified and skeletonized the CHA, to investigate whether the CHA was affected by tumor invasion. The CHA was absent from the cranial side of the pancreas. The artery was encircled behind the SMV and in the hepatoduodenal ligament. The aberrant hepatic artery was not invaded by the tumor, and it was carefully preserved while the right gastroepiploic artery and the pancreatic branches were ligated and divided (Figure 4A). After these anatomical variations of the vessels were clearly delineated, the other procedures were performed using the major vessels as landmarks, as in Case 1.

The procedures around the SMA-plexus and the IPDA were performed after the jejunum was transected and the jejunal mesentery near the ligament of Treitz was divided. The jejunum was passed underneath the SMV over to the left side of the abdomen. The surgeon retracted the SMV and PV with the left hand, and he resected the neural plexus connecting around to the SMA and divided the IPDA from the caudal side with support from the assistants. The surgeon used double-armed sutures during the duct-to-mucosal anastomosis of the anterior row in the pancreaticojejunostomy. He stood on the right side of the patient in all surgical procedures.

The operation time was 659 min, and the intraoperative blood loss was 350 ml. Histopathological examination of the tumor revealed a well-differentiated adenocarcinoma of the bile duct that was classified as T1N0M0 stage IA according to the AJCC/UICC TNM staging system [16] (Figure 4B). The biliary, pancreatic, duodenal, and retroperitoneal margins were free of tumor. The patient’s postoperative course was complicated by a pancreatic fistula (ISGPF-grade-B) without any symptoms of infection. The patient was discharged after the leakage was ameliorated with drainage.

## Discussion

Situs inversus totalis denotes complete right-left inversion of thoracic and abdominal viscera. Predisposition to SIT is a rare congenital anomaly [1]. Although there are some case reports of SIT with various types of cancer, the pathogenic mechanisms of SIT, and the association between SIT and neoplasia have not been well elucidated.

Situs inversus totalis occurs frequently with Kartagener syndrome, anomalies of the hepatobiliary system [19], and cardiovascular malformations [9]. In a study by Mayo and Rice [19], 7 of 76 patients with SI were found to have abnormalities of the biliary tract or gallbladder. Variations in the hepatic artery have also been reported. Uemura *et al.*[9] reported variations in the hepatic artery in three of six patients with SI who underwent hepatectomy.

For hepatobiliary-pancreatic surgery, advanced diagnostic imaging techniques, such as three-dimensional CT, are highly recommended whenever available [20-22]. In the present cases of pancreaticoduodenectomy, three-dimensional reconstructed imaging by MD-CT data, especially non-invasive angiography, enabled understanding of the regional anatomy and allowed meticulous preoperative planning. Each surgical procedure was performed while checking the positions of the major vessels one by one and taking extreme care with every approach. This allowed the patients to undergo pancreaticoduodenectomy safely and effectively. Both patients had an anomaly of the hepatic artery. In Case 2, the CHA arose from the SMA and ran through behind the SMV. This anomaly of the hepatic artery belonged to type VI of Adachi’s classification and type IX of Michels’ classification [17,18]. This type of anomaly is found in 4.5% of all individuals [18]. Particularly in Case 2, the preoperative imaging of the vascular arrangement helped avoid intraoperative injuries and postoperative complications associated with the unusual arrangement of the hepatic artery.

Several malformations of transposed organs and vascular anatomical variations associated with SIT complicate surgical management. Previous authors have reported performing pancreaticoduodenectomy in patients with SI (Table 1) [10-15]. Almost all authors have suggested that detailed preoperative imaging studies are needed to identify the presence or absence of associated anatomic abnormalities and to perform the surgical procedure safely. However, few reports, other than ours, have described operative blood loss and operative duration (Table 1). Furthermore, this is the first report of pancreaticoduodenectomy in the patients with SIT describing detailed surgical procedure and techniques.

**Table 1:** Previous reports of pancreaticoduodenectomy in patients with situs inversus (totalis)

**Reference**	**Age/sex**	**Malignancy**	**Blood loss (ml)**	**Operation time (min)**
[10]	44/F	Ampulla of Vater	NA	NA
[11]	65/M	Bile duct	1596	522
[12]	67/M	Pancreas head	NA	NA
[13]	48/F	Pancreas head	NA	NA
[14]	63/M	Pancreas head	865	635
[15]	33/M	Bile duct	NA	NA
Present cases	74/M	Bile duct	600	564
	67/M	Bile duct	350	659

NA, not available.

There was some confusion about the location of the abdominal viscera in the early phases of the operation, but we adapted to the mirror image of the standard procedure during the operation because the operative procedure was planned based on the preoperative imaging studies. During most of the surgery in the present cases, the operator positioned himself on the right side of the patient, as usual. Two reports described the surgeon’s position during pancreaticoduodenectomy in patients with SIT [11,14]. The authors reported that the surgeon stood on the right side of the patient during the operation, as in the present cases. In the present Case 1, the surgeon stood on the left side of the patient in only two situations, during excision of the SMA-plexus and during pancreaticojejunostomy. Depending on various situations, the surgeon should change position to ensure an adequate surgical procedure. Furthermore, the assistants should support the operation appropriately because the surgeon is required to 'reverse’ the handling technique. Table 2 shows difficulties and technical issues in pancreaticoduodenectomy in patients with SIT (Table 2).

**Table 2:** Difficulties and technical issues in pancreaticoduodenectomy in patients with situs inversus (totalis)

**Step**	**Procedure**	**Difficulties**	**Appropriate techniques**
Extirpation	Surgeon’s position	Mirror-imaged transposition of abdominal viscera	The surgeon stands on the right side of the patient.
	Vessel control	Vascular anomalies	Careful preoperative anatomic assessment by detailed imaging studies is needed.
			It is helpful to encircle the major vessels and use them as landmarks, to avoid any errors.
	Handling technique	Left-to-right reversal of the standard procedure	Reconstructed imaging by MD-CT is useful for preoperative planning.
			The surgeon changes position depending on various situations.
			The assistants adequately support the operation.
Reconstruction	Pancreaticojejunostomy	Reversed suture technique	The surgeon decides the best position for suturing.
	Hepaticojejunostomy		The doubly armed sutures are useful in pancreaticojejunostomy.
	Duodenojejunostomy		
Drainage	Route	Same routes as the standard procedure	Two closed-system drainages are placed in the foramen of Winslow and around the pancreaticojejunal anastomosis.

## Conclusions

In conclusion, advanced diagnostic imaging techniques are very important for careful preoperative planning and to prevent misunderstanding of the arrangement of the abdominal viscera. These techniques facilitate adaptation to the mirror image of the standard procedure and help avoid intraoperative complications due to the cardiovascular and hepatobiliary malformations associated with SIT. Pancreaticoduodenectomy in patients with SIT can be performed successfully with detailed preoperative assessment, use of effective techniques by the surgeon, and appropriate support by assistants.

## Consent

Written informed consent was obtained from the patients for publication of this case report and any accompanying images. A copy of the written consent is available for review by the editor-in-chief of this journal.

## Abbreviations

AJCC/UICC: American Joint Committee on Cancer/Union for International Cancer Control; BD: Bile duct; CHA: Common hepatic artery; CT: Computed tomography; GDA: Gastroduodenal artery; IPDA: Inferior pancreaticoduodenal artery; ISGPF: International Study Group of Pancreatic Fistula; IVC: Inferior vena cava; MD-CT: Multidetector-row CT; PHA: Proper hepatic artery; PV: Portal vein; SI: Situs inversus; SIT: Situs inversus totalis; SMA: Superior mesenteric artery; SMV: Superior mesenteric vein.

## Competing interests

The authors declare that they have no competing interests.

## Authors’ contributions

YK and MI (Imamura M) performed the operation. DK, MU, MI (Ishii M), MM, MK, TM, and KH participated in the operation, designed the study and helped to collect data. DK, KY and KH wrote the paper together. All authors read and approved the final manuscript.
